# Complement-Mediated Thrombotic Microangiopathy Related to COVID-19 or SARS-CoV-2 Vaccination

**DOI:** 10.1016/j.ekir.2023.05.010

**Published:** 2023-05-22

**Authors:** Christof Aigner, Martina Gaggl, Sophie Schmidt, Renate Kain, Nicolas Kozakowski, André Oszwald, Zoltán Prohászka, Raute Sunder-Plassmann, Alice Schmidt, Gere Sunder-Plassmann

**Affiliations:** 1 Division of Nephrology and Dialysis, Department of Medicine III, Medical University of Vienna, Vienna, Austria; 2 Department of Pathology, Medical University of Vienna, Vienna, Austria; 3 Research Laboratory, Department of Internal Medicine and Hematology, and Research Group for Immunology and Hematology, Semmelweis University- EötvösLoránd Research Network (Office for Supported Research Groups), Budapest, Hungary; 4 Genetics Laboratory, Department of Laboratory Medicine, Medical University of Vienna, Vienna, Austria

**Keywords:** COVID-19, genetic renal disease, hemolytic uremic syndrome, SARS-CoV-2, thrombotic microangiopathy, vaccination

## Abstract

**Introduction:**

Infectious diseases and vaccinations are trigger factors for thrombotic microangiopathy. Consequently, the COVID-19 pandemic could have an effect on disease manifestation or relapse in patients with atypical hemolytic syndrome/complement-mediated thrombotic microangiopathy (aHUS/cTMA).

**Methods:**

We employed the Vienna TMA cohort database to examine the incidence of COVID-19 related and of SARS-CoV-2 vaccination-related relapse of aHUS/cTMA among patients previously diagnosed with aHUS/cTMA during the first 2.5 years of the COVID-19 pandemic. We calculated incidence rates, including respective confidence intervals (CIs) and used Cox proportional hazard models for comparison of aHUS/cTMA episodes following infection or vaccination.

**Results:**

Among 27 patients with aHUS/cTMA, 13 infections triggered 3 (23%) TMA episodes, whereas 70 vaccinations triggered 1 TMA episode (1%; odds ratio 0.04; 95% CI 0.003–0.37, *P* = 0.01). In total, the incidence of TMA after COVID-19 or SARS-CoV-2 vaccination was 6 cases per 100 patient years (95% CI 0.017–0.164) (4.5/100 patient years for COVID-19 and 1.5/100 patient years for SARS-CoV-2 vaccination). The mean follow-up time was 2.31 ± 0.26 years (total amount: 22,118 days; 62.5 years) to either the end of the follow-up or TMA relapse (outcome). Between 2012 and 2022 we did not find a significant increase in the incidence of aHUS/cTMA.

**Conclusion:**

COVID-19 is associated with a higher risk for aHUS/cTMA recurrence when compared to SARS-CoV-2 vaccination. Overall, the incidence of aHUS/cTMA after COVID-19 infection or SARS-CoV-2 vaccination is low and comparable to that described in the literature.

## Graphical abstract

**Figure:**
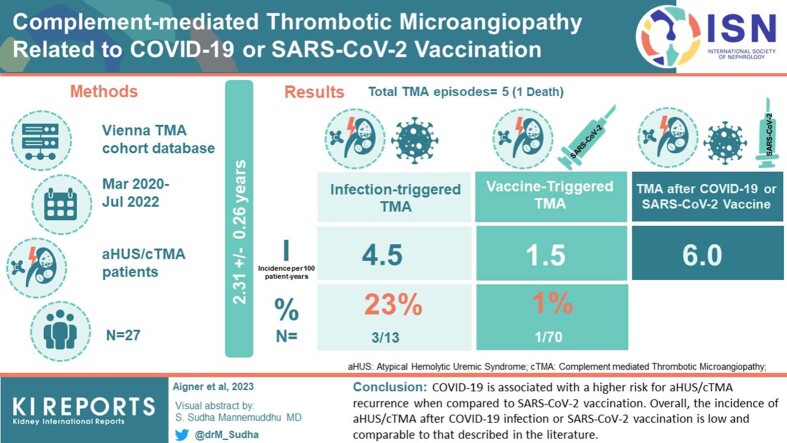

Complement dysregulation and activation is involved in the pathogenesis of COVID-19 that is due to infection with SARS-CoV-2. Elevated plasma levels of the terminal complement complex (sC5b-9) have been reported in a large number of COVID-19 patients and are associated with a more severe course of the disease.^1^

Another condition in which complement dysregulation and activation plays a central role is aHUS. Here, patients suffer from kidney injury related to cTMA. Of these patients, 60% to 80% carry rare, predisposing complement gene variants and certain trigger factors, including infectious diseases or vaccinations, that can induce the disease.^2^ Therefore, COVID-19 and SARS-CoV-2 vaccination could be factors triggering recurrent aHUS/cTMA in vulnerable patients.^3^, ^4^, ^5^

We therefore addressed this clinically important issue using data derived from the Vienna TMA cohort database and examined the impact of the COVID-19 pandemic on disease manifestation in patients diagnosed with cTMA/aHUS. We hypothesized that COVID-19 and SARS-CoV-2 vaccination both could activate the complement system resulting in aHUS/cTMA episodes in these patients.

The specific objective of this study was therefore to examine whether COVID-19 or SARS-CoV-2 vaccination pose a greater risk for an aHUS/cTMA episode in patients with an established diagnosis of aHUS/cTMA before begin of the COVID-19 pandemic.

## Methods

### Study Design

In this cohort study, we examined the frequency of aHUS/cTMA episodes related to COVID-19 and SARS-CoV-2 vaccination among patients with an established diagnosis of aHUS/cTMA before the beginning of the COVID-19 pandemic (STROBE Statement). The institutional review-board of the Medical University of Vienna approved the study (unique IRB identifier: 1368/2014). All patients provided written informed consent and the study was conducted in accordance with the principles of the Declaration of Helsinki.

### Setting

We used the Vienna TMA cohort database, which was established in 2014 at the Division of Nephrology and Dialysis, Department of Medicine III of the Medical University of Vienna. It includes all patients that presented with TMA irrespective of the underlying disease or complement disorder as well as patients with other complement-mediated kidney diseases (such as C3 glomerulonephritis) under the clinical care of our institution, which has a catchment area in Eastern Austria of about 4 million inhabitants.^6^^,^^7^ For this study, only patients with a previously established diagnosis of aHUS/cTMA were included. The study period began in March 2020 with a follow-up until July 2022, representing the first 29 months of the COVID-19 pandemic in Austria.

### Study Participants

Prevalent patients with a history of aHUS/cTMA and active clinical surveillance at the beginning of the COVID-19 pandemic qualified for inclusion in this analysis. Follow-up during the study period was done in the Nephrology outpatient service of our institution.

### Variables

SARS-CoV-2 infections were recorded in case of a positive result of a real-time quantitative polymerase chain reaction test. SARS-CoV-2 vaccinations are free of charge in Austria with specific recommendations for individuals at high-risk for severe COVID-19, such as patients with aHUS/cTMA. A TMA episode was defined either by the presence of specific laboratory signs and/or of TMA on a kidney biopsy.

### Data Sources and Measurements

Demographic data, clinical data, and data on COVID-19 and SARS-CoV-2 vaccinations were retrieved from electronic and paper-based health care records, as well as personal interviews with all patients in August and September 2022.

With regard to COVID-19 in Austria, both nasal/pharyngeal swabs performed by health care professionals and at-home gargling tests are common methods of sampling. A real-time quantitative polymerase chain reaction test result with a cycle threshold of less than 30 was deemed positive for the presence of SARS-CoV-2.

For SARS-CoV-2 vaccination, the date, and the type of vaccine are documented for all inhabitants of Austria in a single, national health care database, which can be accessed by treating physicians.

A TMA episode was defined either by the presence of systemic signs of TMA (mechanical hemolysis, thrombocytopenia, and acute kidney injury; thrombotic thrombocytopenic purpura was ruled out by analysis of ADAMTS13 and autoimmune hemolysis was ruled out by performing Coombs tests) or by the presence of signs of TMA (thrombi in glomerular capillaries or arterioles, mesangiolysis, endothelial swelling, double contouring of basement membranes, and multilayering of arterioles) on a kidney biopsy.^8^ An association with COVID-19 or SARS-CoV-2 vaccination was considered if any signs or symptoms of TMA occurred within 8 weeks (which was the maximum interval that was used in the literature) of the respective event and no other triggering factor could be identified.

All standard laboratory work-up was performed at the Department of Laboratory Medicine, Medical University of Vienna. Complement-specific analyses were performed at the Research Laboratory, Department of Internal Medicine and Hematology 3rd Department of Internal Medicine and Hematology, and Research Group for Immunology and Hematology, Semmelweis University–EötvösLoránd Research Network (Office for Supported Research Groups), Budapest, Hungary.^9^ Genetic analyses of *CFH, CFI, CD46, C3, CFB, THBD*, and *CHFR-5* were performed by the same 2 institutions for routine clinical purposes.^7^

### Statistical Methods

The data are presented as count and frequency, as mean and standard deviation, or as median and interquartile range, as appropriate. Fisher exact test was used to calculate differences in the proportions of categorical variables and odds ratios. We calculated incidence rates (events/person-year × 100) and respective 95% CIs. Patients could be exposed (SARS-CoV-2 infection or vaccination) multiple times over the follow-up time. Each time the exposure status changed patients were censored and reentered the cohort to contribute person-time to the updated exposure status. Follow-up time ended and patients were censored and not reentered into the cohort after the outcome (TMA relapse) was reached. After July 2022 all patients who had not reached the outcome yet were administratively censored. We used Cox proportional hazards regression to estimate hazard ratios. We speculated that eculizumab or ravulizumab therapy would have an effect on the probability of TMA episodes and therefore we introduced it as an interaction variable. We used a 95% CI, and a 2-sided *P*-value of < 0.05 was considered significant. Incidences were compared with descriptive statistics (median and CI) and the Wilcoxon test. We used MS Excel and GraphPad Prism 9 for data management and analysis and GraphPad Prism 9 for the generation of figures.

## Results

### Participants

**Figure 1:**
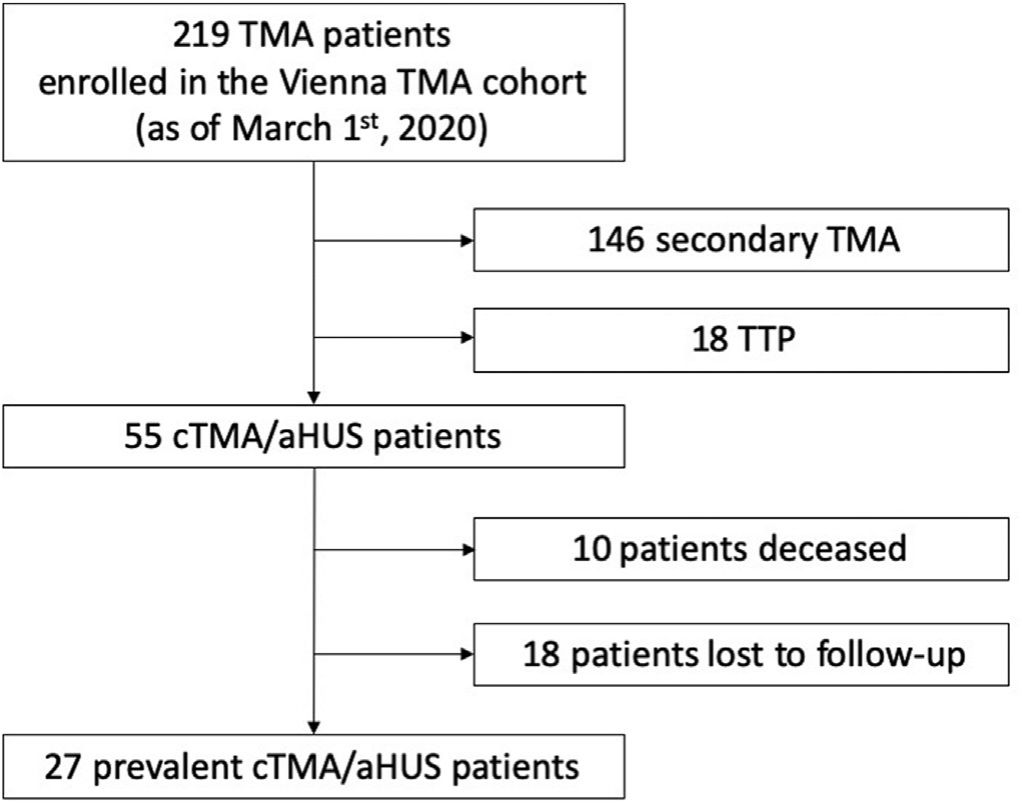
Patient disposition. aHUS, atypical hemolytic syndrome; cTMA, complement-mediated thrombotic microangiopathy; TMA, thrombotic microangiopathy; TTP, thrombotic thrombocytopenic purpura.

As of March 2020, the Vienna TMA cohort comprised 217 patients with a TMA of any cause, including 55 patients with a diagnosis of aHUS/cTMA. Of those, 27 were in active clinical surveillance at the beginning of the COVID-19 pandemic. The mean follow-up time was 2.31 ± 0.26 years (total amount: 22,118 days; 62.5 years) to either the end of the follow-up or TMA relapse (outcome). The patient disposition is indicated in Figure 1. Demographic data, SARS-CoV-2 vaccination status, as well as COVID-19 episodes are summarized in Table 1.

**Table 1:** Patient characteristics

Characteristic	All patients
Patient number, *N*	27
Female, *n*	19 (70%)
Mean age at the start of the COVID-19 pandemic, years	41.6 ± 15.7
Race	
Caucasian, *n*	23 (85%)
African, *n*	3 (11%)
Asian, *n*	1 (4%)
Maintenance anti C5 treatment any time during pandemic, *n*	8 (30%)
SARS-CoV-2 vaccination, *n*	23 (85%)
1, *n*	1 (4%)
2, *n*	5 (22%)
3, *n*	11 (48%)
4, *n*	4 (17%)
5, *n*	2 (9%)
SARS-CoV-2 infection, *n*	13 (48%)
Wuhan strain, *n*	1 (4%)
Alpha, *n*	0
Delta, *n*	1
Any Omicron variant, *n*	12 (44%)
TMA episode during pandemic, any cause	5 (19%)
TMA episode after SARS-CoV-2 infection	3 (11%)
TMA episode after SARS-CoV-2 vaccination	1 (4%)

anti C5 treatment, maintenance eculizumab or ravulizumab; SARS-CoV-2, severe acute respiratory distress syndrome corona virus 2; TMA, thrombotic microangiopathy, *n* = number.

### SARS-CoV-2 Vaccination

In total, 23 (85%) patients were vaccinated with 70 doses against COVID-19 (any vaccine), with 1 patient receiving 1 (4%) dose, 5 (22%) patients receiving 2 doses, 11 (48%) patients were vaccinated 3 times, and 4 (17%) and 2 (9%) patients received 4 and 5 doses, respectively. The most widely used vaccine was Comirnaty (Pfizer, New York City, NY; *n* = 49, 70%) followed by Spikevax (Moderna, Cambridge, MA; *n* = 17, 24%) and Vaxzevria (Astra Zeneca, Cambridge, UK; *n* = 4, 5%). Four patients (15%) refused to be vaccinated.

### COVID-19

We recorded a total of 13 SARS-CoV-2 infections in 13 patients, all of which were clinically mild. Most of the infections (11; 84.6%) occurred during the SARS-CoV-2 waves of the Omicron variants in Austria, which were the dominant variants from the end of the year 2021 onwards.

### TMA Episodes During the Pandemic

**Figure 2:**
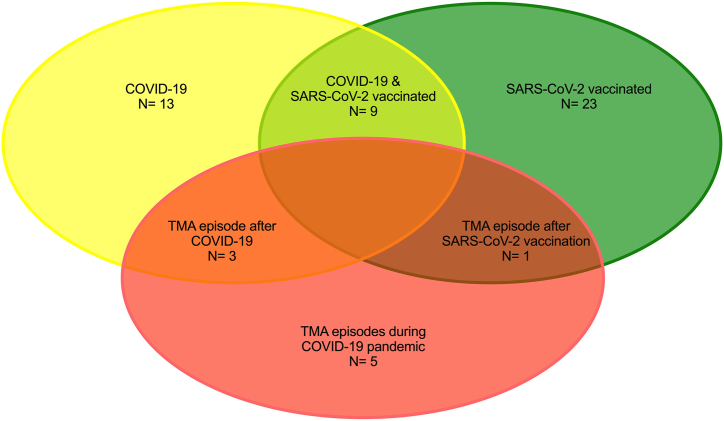
TMA relapses triggered by SARS-CoV-2 vaccination or COVID-19. TMA, thrombotic microangiopathy.

**Figure 3:**
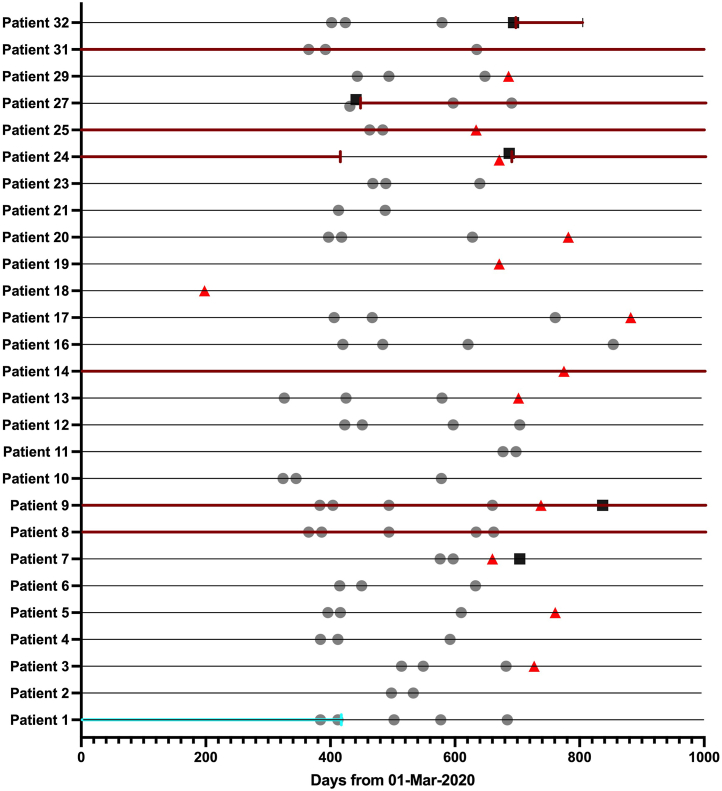
SARS-CoV-2 vaccinations, COVID-19, TMA episodes and therapy in all patients during the COVID-19 pandemic. Turquoise arrows: plasma therapy; red arrows: eculizumab or ravulizumab; gray dots: vaccinations; black squares: COVID-19; red triangles: TMA episode. Black lines indicate time of follow-up.

Between March 2020 and July 2022, we recorded a total of 5 TMA episodes in 27 patients enrolled in the Vienna TMA cohort (Figure 2). The individual course of all 27 patients, including SARS-CoV-2 vaccinations, COVID-19 episodes, TMA episodes during the pandemic, and complement therapies are presented in Figure 3. One patient died because of a preexisting malignant disease. Four (80%) of these 5 TMA episodes were related to COVID-19 (*n* = 3; patient 7, 9, 24) or SARS-CoV-2 vaccination (*n* = 1; patient 27). Case vignettes of these 4 TMA cases are provided in the Supplementary Material. One other case was an aHUS/cTMA relapse most likely related to a fatal malignant disease (patient 32).

One patient showed systemic signs of TMA after COVID-19 (patient 24). The 3 other patients with a history of kidney transplantation showed no signs of microangiopathic hemolysis but worsening of graft function (patient 7, COVID-19; patient 27, SARS-CoV-2 vaccination with Spikevax [Moderna, Cambridge, MA]), and a sharp rise in proteinuria (patient 9, COVID-19), respectively. A biopsy of the kidney graft showed TMA in all 3 transplant cases (patient 7, 9, and 27). Details of these 4 patients are indicated in Table 2.

**Table 2:** Characteristics of patients with COVID-19 or SARS-CoV-2 vaccination associated TMA

						TMA diagnosis						
Patien number	Sex	Age	Event	Time to TMA from event^b^	Native kidney; kindey graft	SCr	PLT	LDH	UPCR	Kidneybiopsy	TMA treatment	Outcome3 mo
7^a^	M	34	COV	44	G	6.28	207	252	4746	active TMA	5× PLEX	SCr 6.78
9^a^	F	32	COV	28	G	1.54	355	203	3425	TMA vs. ABMR	on antiC5	SCr 1.66
24^a^	F	21	COV	16	N	18.96	124	422	n.a.	n.a.	anti C5	chronic HD
27^a^	M	67	VAC	10	G	2.54	132	277	3246	relapsing TMA	antiC5	SCr 2.63

ABMR, antibody mediated rejection; anti C5, eculizumab or ravulizumab therapy; *CFH*, complement factor H; COV, coronavirus disease 19; F, female; G, kidney graft; HD, hemodialysis; LDH, lactate dehydrogenase (reference range: <250 U/l); M, male; N, native kidney; n.a., not available; PLEX, plasma exchange; PLT, platelet count (reference range: 150-350 G/L); SCr, serum creatinine in mg/dl; TMA, thrombotic microangiopathy; UPCR, urinary protein to creatinine ratio (reference range: <200 mg/g); VAC, SARS-CoV-2 vaccination.

a TMA relapse.

b In days.

The complement profile of all 4 patients with TMA related to COVID-19 or SARS-CoV-2 vaccination is presented in Table 3. All 4 patients showed an elevated serum concentration of the terminal complement complex sC5b-9. One patient (patient 27) that suffered a TMA episode after a SARS-CoV-2 vaccination received a further immunization with an mRNA vaccine under ravulizumab therapy and consequently showed no further signs of TMA. During the study period, 8 (29.6%) patients received treatment with eculizumab or ravulizumab at different times during follow-up. Two patients (7%) received plasma therapy during the pandemic (Figure 3). Three patients had mild COVID-19 and 5 patients received SARS-CoV-2 vaccinations while on eculizumab or ravulizumab, respectively.

**Table 3:** Complement serology at TMA episode

Patient	7	9	24	27	Reference range	
TCA, classical pathway	84	16^a^	63	76	48–103 CH50/ml	
TCA, alternative pathway	110	1^a^	68^a^	112	70–125 %	
Complement C3	1.39	1.13	0.78^a^	1.0	0.9–1.8 g/l	
Complement C4	0.64	0.28	0.39	0.38	0.15–0.55 g/l	
Factor H ag	775	231^a^	333	366	250–880 mg/l	
Complement factor I ag	111	59^a^	74	90	70–130%	
Complement factor B ag	135	76	106	82	70–130%	
Anti-factor H IgG autoantibody	25	n.a.	n.a.	18	<110 AU/ml	
sC5b-9 (TCC)	n.a.	363^a^	384^a^	436^a^	110–252 ng/ml	
C1q ag	100	56	69	115	60–180 mg/l	
Anti-C1q IgG autoantibody	1	n.a.	n.a.	2	<52 U/ml	
ADAMTS13 activity	84	n.a.	65	59	67–150 %	

ADAMTS13, a disintegrin and metalloproteinase with a thrombospondin type 1 motif, member 13; ag, antigen; n.a., not available; TCA, total complement activity; TCC, terminal complement complex.

a Values that do not lie within the reference range and are clinically relevant.

### Incidence of TMA Episodes Associated With COVID-19 or SARS-CoV-2 Vaccination

In total, 13 infections triggered 3 (23%) TMA episodes, whereas 70 vaccinations triggered 1 TMA episode (1%; odds ratio 0.04; 95% CI 0.003–0.37, *P* = 0.01). Two vaccinations were administered after the outcome event under therapy with ravulizumab and no further TMA episodes were registered.

In total, the incidence of TMA after COVID-19 or SARS-CoV-2 vaccination was 6 cases per 100 patient years (95% CI 0.017–0.164) (4.5/100 patient years for COVID-19 and 1.5/100 patient years forSARS-CoV-2 vaccination).

The hazard ratio for a TMA episode after COVID-19 when compared to SARS-CoV-2 vaccination was 7.1 (95% CI 0.56–164.4; *P* = 0.1303) . Ongoing eculizumab/ravulizumab treatment had no modifying effect (*P* > 0.99) on the incidence of TMA episodes associated with COVID-19 or SARS-CoV-2 infections in this cohort.

### Incidence of aHUS/cTMA Before and During the COVID-19 Pandemic

Between 2014 and 2022, a total of 31 patients were diagnosed with cTMA/aHUS at our center. Specifically, 10, 8, and 13 patients were diagnosed between 2014 and 2016, 2017 and 2019, and 2020 and 2022, respectively (median 10; 95% CI 8–13; *P* = 0.25; Supplementary Figure S1).

## Discussion

This first single-center cohort study investigating the association of COVID-19 and SARS-CoV-2 vaccination with thrombotic microangiopathies during the first 2.5 years of the COVID-19 pandemic demonstrates a higher risk of relapse in patients previously diagnosed with aHUS/cTMA following infection compared to vaccination.

The molecular mechanisms of COVID-19 or SARS-CoV-2 vaccination for the development of TMA include a heightened proinflammatory state with disturbances in complement activation and the coagulation cascade.^1^^,^^10^ Viral infections represent rare but well-established trigger factors for aHUS/cTMA.^11^ Direct toxicity of HIV, CMV, HHV6, varicella, and parvovirus B19 to endothelial cells, as well as unmasking of the cryptic Thomsen-Friedenreich antigen on red blood cells and endothelial cells by H1N1 influenza neuraminidase, has been implicated in the pathogenesis of aHUS/cTMA.^11^, ^12^, ^13^ Similarly, in some case reports, SARS-CoV-2 infection, which can result in profound complement activation, was shown to trigger aHUS/cTMA, with preponderance of patients with genetic causes or risk factors for aHUS/cTMA.^14^, ^15^, ^16^, ^17^, ^18^, ^19^ Furthermore, COVID-19 was shown to lead to aHUS/cTMA relapses in patients with an established diagnosis of aHUS/cTMA.^20^, ^21^, ^22^ Several studies have looked at kidney biopsies in patients with COVID-19. The most common findings were collapsing glomerulopathy and acute tubular injury. Thrombotic microangiopathy was diagnosed in less than 5% of biopsies, making it a rare type of kidney disease diagnosed in association with COVID-19.^23^, ^24^, ^25^, ^26^, ^27^, ^28^, ^29^ In the 2 largest studies, Pacheco *et al.*^24^ and May *et al.*^23^ described TMA in 3.6% (*n* = 12) and 2.1% (*n* = 5) of 331 and 250 kidney biopsies, respectively. In our study of patients with aHUS/cTMA with a follow-up during the first 2.5 years of the COVID-19 pandemic, we observed 3 recurrent aHUS/cTMA episodes related to mild COVID-19 in 2 kidney transplant recipients with a primary diagnosis of aHUS/cTMA and in 1 patient with a history of pregnancy associated aHUS/cTMA. Notably, not all patients presented with systemic signs of TMA, but were diagnosed by the means of kidney biopsy.

COVID-19 is now well-established as a trigger factor for TMA; however, reports linking SARS-CoV-2 vaccination to aHUS/cTMA are rare.^14^ The first reported cases of TMA triggered by BNT162b2 from our group or ChAdOx1 nCoV-19 by Ferrer *et al.*^4^ were succeeded by other reports on the association of mRNA-based vaccines *(n =* 5) or adenoviral vector-based vaccines (*n* = 2).^3^, ^4^, ^5^^,^^30^^,^^31^ In the study of Bouwmeester *et al.*,^31^ an additional retrospective analysis of 73 vaccinations among 29 patients with aHUS, including 21 kidney transplant recipients, found no evidence of recurrent TMA episodes triggered by vaccination. In this context, Pacheco *et al.*^24^ summarized published data on kidney biopsies of patients with postvaccination kidney injury and did not refer to any case of aHUS/cTMA. Nevertheless, as of November 27, 2022, the Vigibase database reported 28 cases of aHUS related to SARS-CoV-2 vaccination.^32^ Considering the approximately 13 billion doses of SARS-CoV-2 vaccines that have been administered to date, aHUS/cTMA indeed is a very rare potentially adverse event following vaccination.^33^ A case series in patients with immune-mediated thrombotic thrombocytopenic purpura showed a risk of relapse after administration of an mRNA-based SARS-CoV-2 vaccination (5 of 32 vaccinated patients) and further suggested close monitoring for a relapse in those patients.^34^ Furthermore, another manuscript reported 10 cases of immune-mediated thrombotic thrombocytopenic purpura with 7 new-onset cases and 3 relapses; and therefore, immune-mediated thrombotic thrombocytopenic purpura has to be included in the differential diagnoses of hemolysis and neurologic symptoms in the setting of SARS-CoV-2 vaccination.^35^ On the contrary, no elevated risk of relapse after SARS-CoV-2 vaccination has been reported in patients suffering from congenital thrombotic thrombocytopenic purpura.^36^

In our study, 1 of the 27 patients experienced a recurrence of aHUS/cTMA after vaccination with mRNA-1273. In comparison to aHUS/cTMA associated with COVID-19, SARS-CoV-2 vaccination was associated with lower odds for a recurrence of aHUS/cTMA. This is in line with a nationwide survey in Switzerland, which included incidence data of glomerulonephritis for 7.1 million inhabitants, that recently showed that mRNA-based vaccines do not increase the risk for new-onset glomerulonephritis.^37^ Equally, the incidence of aHUS/cTMA episodes did not change significantly in our center during the last decade in the years before and after the begin of the pandemic (Supplementary Figure S1).

A limitation of our study is the sample size, which is counterbalanced by the complete documentation of patients with aHUS/cTMA enrolled in the Vienna TMA cohort. Assertion bias for SARS-CoV-2 vaccination status can be ruled out in our study because of the nationwide documentation in electronic health care records in our country. With regard to COVID-19, real-time quantitative polymerase chain reaction testing for SARS-CoV-2 was a free of charge and a low-threshold test was routinely used for screening in asymptomatic individuals and in case of flu-like symptoms. Further, since the onset of the pandemic, all hospitalized patients of our institution and of other hospitals are routinely tested at least once per week for the presence of SARS-CoV-2. Because we have done personal interviews with all the participants at the end of the observation period, we do not believe that we have missed mild to severe COVID-19 episodes or hospitalizations for TMA in other hospitals among study participants. Further, an individual recall bias regarding COVID-19 is unlikely given the global attention paid for the pandemic. We do not routinely test for anti-SARS-CoV-2 nucleocapsid protein antibodies in nontransplant patients, which could have provided positive proof of further asymptomatic cases. Obviously, we could not rule out subclinical TMA episodes related or unrelated to COVID-19 or SARS-CoV-2 vaccination.

In conclusion, COVID-19 is associated with a higher risk for aHUS/cTMA recurrence when compared to SARS-CoV-2 vaccination, but overall, the incidence of aHUS/cTMA after COVID-19 or SARS-CoV-2 vaccination is low.

## Disclosure

All the authors declared no competing interests.

## Supplementary Material

Supplementary File (PDF)

**Case Vignettes**

**Figure S1.** Number of incident aHUS/cTMA cases per year.

**STROBE Statement.**
