# Supplementary material for: Complement-Mediated Thrombotic Microangiopathy Related to COVID-19 or SARS-CoV-2 Vaccination
Source: Kidney Int Rep. 2023 May 22;8(8):1506–13. doi: 10.1016/j.ekir.2023.05.010 (PMC10201914; doi:10.1016/j.ekir.2023.05.010)

STROBE Statement—checklist of items that should be included in reports of observational studies

	Item No.	Recommendation	Page No.	Relevant text from manuscript
**Title and abstract**	1	(*a*) Indicate the study's design with a commonly used term in the title or the abstract	2	
		(*b*) Provide in the abstract an informative and balanced summary of what was done and what was found	2	
**Introduction**				
Background/rationale	2	Explain the scientific background and rationale for the investigation being reported	4	
Objectives	3	State specific objectives, including any prespecified hypotheses	4	
**Methods**				
Study design	4	Present key elements of study design early in the paper	5	
Setting	5	Describe the setting, locations, and relevant dates, including periods of recruitment, exposure, follow-up, and data collection	5	
Participants	6	(*a*) *Cohort study*—Give the eligibility criteria, and the sources and methods of selection of participants. Describe methods of follow-up<br>*Case-control study*—Give the eligibility criteria, and the sources and methods of case ascertainment and control selection. Give the rationale for the choice of cases and controls<br>*Cross-sectional study*—Give the eligibility criteria, and the sources and methods of selection of participants	5	
		(*b*) *Cohort study*—For matched studies, give matching criteria and number of exposed and unexposed<br>*Case-control study*—For matched studies, give matching criteria and the number of controls per case		
Variables	7	Clearly define all outcomes, exposures, predictors, potential confounders, and effect modifiers. Give diagnostic criteria, if applicable	6	
Data sources/ measurement	8*	For each variable of interest, give sources of data and details of methods of assessment (measurement). Describe comparability of assessment methods if there is more than one group	*6*	
Bias	9	Describe any efforts to address potential sources of bias	14	
Study size	10	Explain how the study size was arrived at	5	

Continued on next page

Quantitative variables	11	Explain how quantitative variables were handled in the analyses. If applicable, describe which groupings were chosen and why	7
Statistical methods	12	(*a*) Describe all statistical methods, including those used to control for confounding	7
		(*b*) Describe any methods used to examine subgroups and interactions	7
		(*c*) Explain how missing data were addressed	7
		(*d*) *Cohort study*—If applicable, explain how loss to follow-up was addressed<br>*Case-control study*—If applicable, explain how matching of cases and controls was addressed<br>*Cross-sectional study*—If applicable, describe analytical methods taking account of sampling strategy	7
		(*e*) Describe any sensitivity analyses	
**Results**			
Participants	13*	(a) Report numbers of individuals at each stage of study—eg numbers potentially eligible, examined for eligibility, confirmed eligible, included in the study, completing follow-up, and analysed	9
		(b) Give reasons for non-participation at each stage	9
		(c) Consider use of a flow diagram	Figure1
Descriptive data	14*	(a) Give characteristics of study participants (eg demographic, clinical, social) and information on exposures and potential confounders	9
		(b) Indicate number of participants with missing data for each variable of interest	9
		(c) *Cohort study*—Summarise follow-up time (eg, average and total amount)	9
Outcome data	15*	*Cohort study*—Report numbers of outcome events or summary measures over time	*9&10*
		Case-control study—Report numbers in each exposure category, or summary measures of exposure	
		Cross-sectional study—Report numbers of outcome events or summary measures	
Main results	16	(*a*) Give unadjusted estimates and, if applicable, confounder-adjusted estimates and their precision (eg, 95% confidence interval). Make clear which confounders were adjusted for and why they were included	11
		(*b*) Report category boundaries when continuous variables were categorized	
		(*c*) If relevant, consider translating estimates of relative risk into absolute risk for a meaningful time period	

Continued on next page

Other analyses	17	Report other analyses done—eg analyses of subgroups and interactions, and sensitivity analyses	11
**Discussion**			
Key results	18	Summarise key results with reference to study objectives	12
Limitations	19	Discuss limitations of the study, taking into account sources of potential bias or imprecision. Discuss both direction and magnitude of any potential bias	14
Interpretation	20	Give a cautious overall interpretation of results considering objectives, limitations, multiplicity of analyses, results from similar studies, and other relevant evidence	14
Generalisability	21	Discuss the generalisability (external validity) of the study results	14
**Other information**			
Funding	22	Give the source of funding and the role of the funders for the present study and, if applicable, for the original study on which the present article is based	16

*Give information separately for cases and controls in case-control studies and, if applicable, for exposed and unexposed groups in cohort and cross-sectional studies.

Note: An Explanation and Elaboration article discusses each checklist item and gives methodological background and published examples of transparent reporting. The STROBE checklist is best used in conjunction with this article (freely available on the Web sites of PLoS Medicine at http://www.plosmedicine.org/, Annals of Internal Medicine at http://www.annals.org/, and Epidemiology at http://www.epidem.com/). Information on the STROBE Initiative is available at www.strobe-statement.org.

Complement-mediated thrombotic microangiopathy related to COVID-19 or SARS-CoV-2 vaccination: a cohort study

Christof Aigner, MD[1], Martina Gaggl, PhD[1], Sophie Schmidt[1], Renate Kain, PhD[2], Nicolas Kozakowski, MD[2], ZoltánProhászka, PhD[3], Raute Sunder-Plassmann, MD[4], Alice Schmidt, MD[1], Gere Sunder-Plassmann, MD.[1]

[1]Division of Nephrology and Dialysis, Department of Medicine III, Medical University of Vienna, Vienna, Austria.
[2]Department of Pathology, Medical University of Vienna, Vienna, Austria.
[3]Research Laboratory, Department of Internal Medicine and Haematology , and Research Group for Immunology and Haematology, Semmelweis University-EötvösLoránd Research Network (Office for Supported Research Groups), Budapest, Hungary
[4]Genetics Laboratory, Department of Laboratory Medicine, Medical University of Vienna, Vienna, Austria.

Supplemental material

Table of contents — Page

Case vignettes 2

Figure S1 6

Case vignettes

SARS-CoV-2 vaccinations and TMA

Patient 27:

This 67-year-old male patient has a long-standing history of kidney disease caused by aHUS/cTMA, which led to a total of 5 kidney transplantations, the last of which was performed in 2005. Serum creatinine remained stable until around 2020 and then slowly began to rise. A graft biopsy in late January of 2021 showed signs of glomerulopathy with segmental double contouring of the basement membrane, which could either be attributable to chronic TMA or chronic antibody-mediated rejection. However, there were no signs of microvascular inflammation, negativeC4d staining in peritubular capillaries, and no donor-specific antibodies. Four months later (May 2021), the patient came to the emergency department with severe dyspnea 10 days after receiving a first dose of SARS-CoV-2 vaccination (Spikevax, Moderna, Cambridge, MA). The serum creatinine concentration was 2.5mg/dL (baseline around 2mg/dL) and there were no signs of microangiopathic hemolysis. Another graft biopsy showed signs of relapsing thrombotic microangiopathy which prompted initiation of complement blockade with ravulizumab. Additionally, he was diagnosed with non-ST-elevation myocardial infarction shortly after the kidney biopsy and underwent coronary artery bypass graft surgery. At the most recent follow-up, the patient had stable serum creatinine levels around 2.5-3 mg/dL. Genetic testing revealed a heterozygous variant in *CD46* (p.Ala353VaL), a rare combined deletion of *CFHR1* and exon 6 of *CFHR3,* and a homozygous *CFH*-H3 risk haplotype. The patient received one further dose of Spikevax (Moderna, Cambridge, MA) and a booster dose with Comirnaty (Pfizer, New York City, NY) while receiving ravulizumab and showed no further signs of TMA.

COVID-19 and TMA

Patient 7:

This 34-year-old male had a history of end-stage kidney disease caused by aHUS/cTMA in 2011 and received a kidney transplant in 2018 with poor graft function from the beginning. Repeat kidney biopsies until September 2020 did not show any signs of TMA or graft rejection, but did show considerable vascular damage. Shortly after mild COVID-19, he came to our department in December 2021 with a significant increase of serum creatinine from his baseline, but without any serological signs of TMA. Due to sustained worsening of his kidney

function, he then underwent a transplant biopsy, which showed active arteriolar TMA. He was treated with 5 plasma exchanges, but his kidney function did not improve. Because of extensive scarring in the transplant biopsy, therapeutic blockade of terminal complement was not considered. He currently has non-dialysis-dependent CKD stage G5A3T. Genetic analysis revealed no rare variants or risk haplotypes and the complement levels in serum were unremarkable.

Patient 24:

This patient suffered from pregnancy-associated aHUS/cTMA at the age of 20 years. Specific treatment included plasma exchange and complement blockade. She required dialysis for about 10 weeks, and treatment with eculizumab was stopped one year later. At this time, she had stable CKD stage G5A3 with a serum creatinine of 6 mg/dL and no laboratory signs of TMA. Six months later, she had mild COVID-19, and two weeks later she presented with a general feeling of weakness and nausea. The laboratory work-up showed acute-on-chronic kidney failure (serum creatinine 18 mg/dl) and mechanical hemolysis, pointing to a TMA relapse. Additionally, C3 in serum was decreased and sC5b-9 was elevated. We re-established treatment with complement blockade but she remains dialysis dependent since then. Genetic testing did not reveal any rare variants or risk-haplotypes of TMA related genes.

Patient 9:

In 2009, this patient commenced hemodialysis at the age of 19 years because of aHUS/cTMA with no response to therapeutic plasma exchange. Genetic testing showed a pathogenic, heterozygous variant in *CFH* (p.Cys1032*). Deceased donor kidney transplantation was performed in 2013 with preventive plasma therapy, and then complement blockade due to intolerance to plasma infusions. She later had a miscarriage despite eculizumab therapy, but her second pregnancy was successful with intensified complement blockade in the second and third trimester (weekly infusion of 900 mg eculizumab).[11] After that, eculizumab infusions were administered every 4 weeks at a dose of 1200mg. In 2021, her immunosuppression was changed from mycophenolate to azathioprine as soon as she mentioned the desire to get a baby, leaving tacrolimus and prednisolone unchanged. Eculizumab was administered every 2 weeks at a dose of 1200mg after she reported to have

conceived. Proteinuria increased somewhat during the first months of gestation and worsened even up to a protein/creatinine ratio of 17,500 mg/g shortly after an episode of mild COVID-19 in week 30 of pregnancy. She then delivered a healthy child by Cesarean section in week 34 of pregnancy. During pregnancy and after delivery she had no signs of mechanical hemolysis, and hypertension was well-controlled. However, proteinuria and also serum creatinine remained elevated. A biopsy of the kidney graft three months after delivery showed complex lesions suggesting chronic TMA with older arteriolar thrombi and double contouring of basement membranes. Alternatively, chronic-active antibody-mediated rejection may also be involved in the pathophysiology of these kidney lesions. However, we found only minimal microvascular inflammation and negative pericapillary C4d staining. Donor-specific antibodies against DRB5 were detectable with a mean fluorescence intensity over 5000; however, those were already detectable before the beginning of the second pregnancy. At last follow up, 4 months after delivery, her creatinine was 1.4 mg/dL, and the protein/creatinine ratio was 1,925 mg/g. She currently receives immunosuppressive triple therapy including mycophenolate, and ravulizumab for complement blockade.

Figure S1. Number of incident aHUS/cTMA cases per year.

There was no significant difference in the incidence of aHUS/cTMA episodes in the three periods from 2014-2016 (n= 10), 2017-2019 (n= 8), and 2020-2022 (n= 13), p=0.25 by Wilcoxon rank sum test.

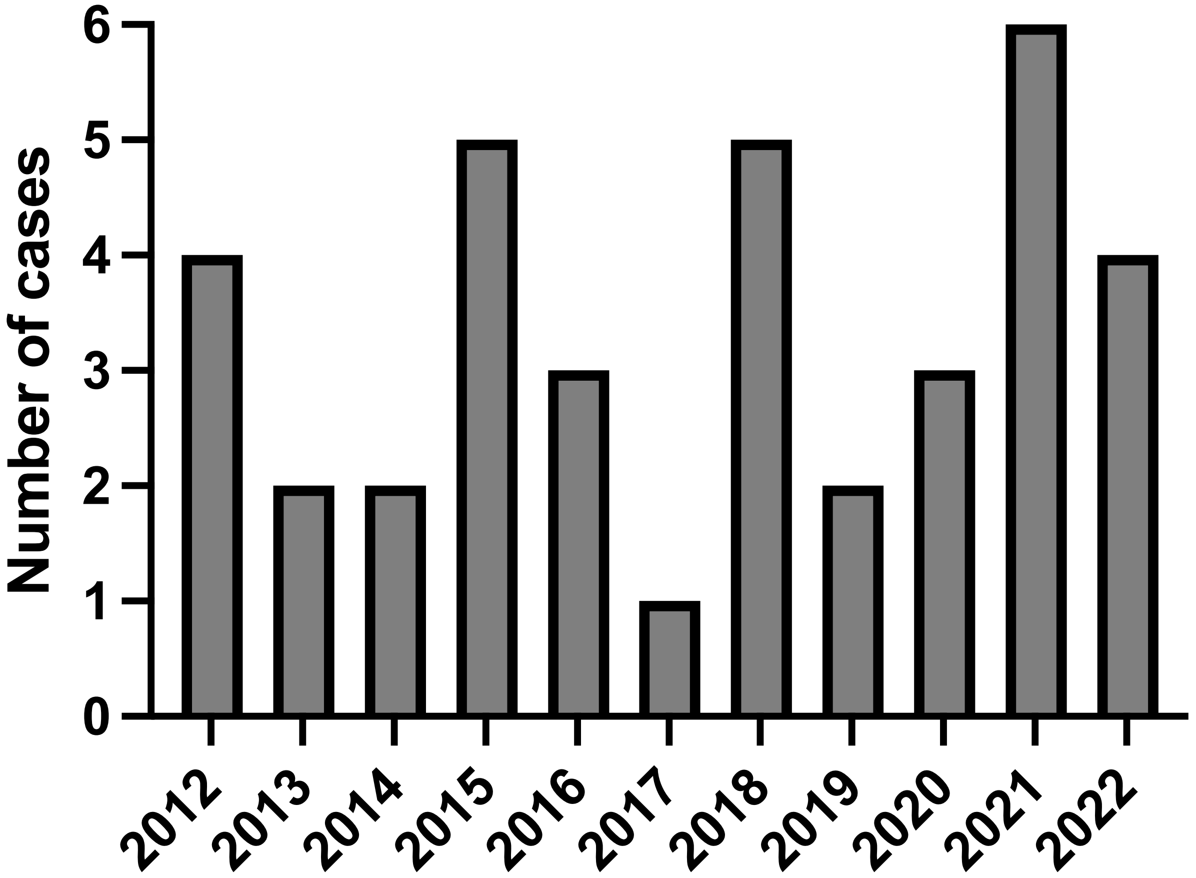

Supplement: Supplementary File (PDF) [file mmc1.pdf]
